# Androgens Increase *lws* Opsin Expression and Red Sensitivity in Male Three-Spined Sticklebacks

**DOI:** 10.1371/journal.pone.0100330

**Published:** 2014-06-25

**Authors:** Yi Ta Shao, Feng-Yu Wang, Wen-Chun Fu, Hong Young Yan, Kazuhiko Anraku, I-Shiung Chen, Bertil Borg

**Affiliations:** 1 Department of Zoology, Stockholm University, Stockholm, Sweden; 2 Taiwan Ocean Research Institute, National Applied Research Laboratories, Kaohsiung, Taiwan; 3 Sensory Physiology Laboratory, Marine Research Station, Academia Sinica, I-lain, Taiwan; 4 Hanse-Wissenschaftskolleg Institute for Advanced Study, Delmenhorst, Germany; 5 Faculty of Fisheries, Kagoshima University, Kagoshima, Japan; 6 Institute of Marine Biology, National Taiwan Ocean University, Keelung, Taiwan; Arizona State University, United States of America

## Abstract

Optomotor studies have shown that three-spined sticklebacks (*Gasterosteus aculeatus*) are more sensitive to red during summer than winter, which may be related to the need to detect the red breeding colour of males. This study aimed to determine whether this change of red light sensitivity is specifically related to reproductive physiology. The mRNA levels of opsin genes were examined in the retinae of sexually mature and immature fish, as well as in sham-operated males, castrated control males, or castrated males implanted with androgen 11-ketoandrostenedione (11 KA), maintained under stimulatory (L16:D8) or inhibitory (L8:D16) photoperiods. In both sexes, red-sensitive opsin gene (*lws*) mRNA levels were higher in sexually mature than in immature fish. Under L16:D8, *lws* mRNA levels were higher in intact than in castrated males, and were up-regulated by 11 KA treatment in castrated males. Moreover, electroretinogram data confirmed that sexual maturation resulted in higher relative red spectral sensitivity. Mature males under L16:D8 were more sensitive to red light than males under L8:D16. Red light sensitivity under L16:D8 was diminished by castration, but increased by 11 KA treatment. Thus, in sexually mature male sticklebacks, androgen is a key factor in enhancing sensitivity to red light via regulation of opsin gene expression. This is the first study to demonstrate that sex hormones can regulate spectral vision sensitivity.

## Introduction

Sexual selection requires the ability to not only find/attract a partner, but also to discern a partner of high quality [1]. Partners are located and attracted via various sensory systems. One or both potential partners emit signals, which can include pheromones, mating calls, or breeding colours. One or both partners recognize and react to the signals using the appropriate sensory system, such as olfaction, hearing, or vision [2]. All other things being equal, the more successful an organism is at sending and/or receiving signals, the more successful it will be at attracting/choosing partners of high quality and/or in high quantity, and thus at disseminating its genes [2]. Sexual selection theory predicts a tight coupling between the evolution of sexual displays and the sensory capabilities of the receiver [1], [3]. Mating signals can be as important for rivals as they are for potential mates [4], [5]. For example, it is important for territorial males to be able to detect the presence of competitors [6], [7].

Generally, sexual signalling changes dramatically with season and state of maturity. Calling attention to oneself outside the breeding season is a waste of energy and can also attract potential predators [8]; seasonal changes in pheromone-detection ability and olfaction sensitivity over the reproductive cycle have been observed in mammals, birds, and teleosts [9]–[11], but such seasonal shifts in the visual system are less well-documented.

The three-spined stickleback, *Gasterosteus aculeatus*, is a well-studied model of reproductive behaviour in fish. A prominent male secondary sexual characteristic is the breeding colour, including blue eyes and a red belly [12]. In nature, the breeding season occurs from late spring to early summer when the photoperiod is long. Many studies have demonstrated that the red colouration is used to attract females [13], and it may be an important signal to competitors as well. It has been often stated that the red belly is a stimulus for male aggressiveness in three-spined sticklebacks. However, the actual data are contradictory, and the majority of studies show no increase in attacks on red models [6]. Nevertheless, it can be predicted that males with a greater ability to detect red light will be more aware of other breeding males, allowing them to react appropriately to competitors.

Using optomotor tests, Cronly-Dillon and Sharma [14] observed higher behavioural red light sensitivity (594 nm), but lower green light sensitivity (516 nm), during summer than during winter in female sticklebacks, but no such differences in males. In contrast, a recent study using an improved optomotor apparatus reported an increase in red light sensitivity (620 nm) in the summer in both sexes [4]. An increase in red light sensitivity in summer does not necessarily indicate a direct role in reproductive physiology; rather, it may be an adaptation to the changing photic environment. In many areas, sticklebacks live in the open sea in winter [15] and migrate to the coast or into freshwater in spring to spawn.

The visual system of fish is composed of photoreceptors, which contain an opsin protein and a light-sensitive chromophore (a vitamin A derivative). There are four major classes of cone opsins, i.e. SWS1, SWS2, RH2, and LWS, and one rod opsin, RH1 [16]. When combined with the chromophore, these opsins form visual pigments with absorbance maxima (λ_max_) in various wavelength regions of the spectrum [16]–[19]. Microspectrophotometry has been used to reveal that sticklebacks possess one class of rod receptor (λ_max_ range: 507–531 nm) and the following four classes of cone photoreceptors: ultraviolet single cones (λ_max_ range: 365–379 nm), blue single cones (λ_max_ range: 434–460 nm), green/red double cones (λ_max_ range: 514–547 nm/566–618 nm), and red/red double cones [18]. Sticklebacks from dystrophic, red light-shifted lakes with visual pigments based on the vitamin A2 chromophore had spectra that were broader and shifted toward longer wavelengths, as compared to ocean (clear water) equivalents with A1-based visual pigments [18]. More clear-cut differences were observed in opsin expression between sticklebacks from clear (ocean and lake) and dystrophic water. Fish from clear waters possess double cones with both LWS and RH2 opsins, one per double cone member. In contrast, fish from red-shifted lakes possess double cones, which are primarily LWS/LWS pairs [18]. In teleosts, both A1/A2 chromophore and opsin gene expression may change at different life stages or seasons, and this may be related to the endocrine system [19]–[23].

Both androgen [24] and oestrogen [25] receptors have been found in the retinae of teleosts, including androgen receptor-β (ARβ) in sticklebacks [26], albeit at low levels. Androgens are well known to stimulate male secondary characteristics and breeding behaviour in the male stickleback [27], and circulating androgen levels peak in the breeding season in both male (mostly 11-ketotestosterone) and female (mostly testosterone) sticklebacks [28]. Therefore, it is possible that androgen and oestrogen act on receptors in the eyes, thereby modulating visual sensitivity and affecting vision-associated reproductive behaviours during the breeding season.

The aim of the present study was to find out whether changes in red light sensitivity in the stickleback are specifically related to reproductive physiology. Male sticklebacks were gonadectomised (this operation could not be performed on females), and the effects of treatment with the androgen 11-ketotestosterone (11 KA) on opsin mRNA expression and spectral sensitivity (as determined using electroretinogram, ERG) were examined.

## Materials and Methods

### Experimental animals

Three-spined sticklebacks were caught at the shore of the Öresund and southern Baltic at the coast of Skåne, south Sweden. Sticklebacks also spawn along these coasts. Three-spined sticklebacks are not a protected or endangered species, and specific permission is not required to collect them. Fish were transported to Stockholm University, after collection on the following dates at the indicated locations (GPS coordinates are given in parentheses): Nov 27^th^–28^th^ 2011 at Skåre (55° 22′ 32.7″ N; 13° 03′ 12.3″ E) and Skanör (55° 24′ 58.1″ N; 12° 49′ 49.6″ E); Feb 24^th^ 2012 at Vikhög (55° 43′ 42.2″ N; 12° 57′ 25.6″ E); and Dec 16^th^ 2012 at Skåre. Based on their size and the season, it was deemed likely that most of the fish had not spawned previously. The animals were housed in 700 or 1200 L aquaria containing clear artificial brackish water (0.5% salinity), which was aerated and filtered, at a density of 0.6 fish/L or less. The aquaria were illuminated by fluorescent tubes, and the light intensity at the water surface was around 100–200 lux. The bottom was covered with sand, and ceramic pots and tubes provided hiding places. All fish were fed primarily on frozen bloodworms, but also with mysids and/or *Artemia* as a carotenoid supplement. Before the experiments, fish were kept at 20°C under a non-stimulatory short day (L8:D16 or initially shorter) photoperiod. For each experiment, all fish were treated identically prior to treatment.

Four sets of experiments were performed. Fish caught in Nov 2011 were used in experiments 1 and 3 and the fish caught in Feb 2012 and Dec 2012 were used in experiments 2 and 4, respectively. Treatments and sample sizes for each experiment are shown in Table 1.

**Table 1:** Experimental treatments.

Sample N	Coloured males	State/Operation	Implant	Photoperiod	Body weight (g)	GSI/KSI(%)
*Experiment 1: Sexual maturity*						
8/Female		Immature	–	Short day	2.15±0.19	3.45±0.54
7/Female		Mature	–	Long day	2.38±0.21	7.86±1.54
8/Male	0	Immature	–	Short day	1.96±0.16	1.08±0.21
8/Male	8	Mature	–	Long day	1.94±0.15	2.86±0.32
*Experiment 2: Gonadectomy under long photoperiod*						
8/Male	6	Sham-operated	–	Long day	1.96±0.14	2.67±0.28
8/Male	0	Castrated	–	Long day	1.71±0.11	1.21±0.19
*Experiment 3: Castration and androgen treatment*						
5/Male	4	Sham-operated	Empty	Long day	2.05±0.17	2.77±0.23
5/Male	0	Castrated	Empty	Long day	1.74±0.09	1.11±0.24
5/Male	4	Castrated	11 KA	Long day	1.69±0.12	2.99±0.24
5/Male	0	Sham-operated	Empty	Short day	1.99±0.14	1.36±0.34
5/Male	0	Castrated	Empty	Short day	1.73±0.12	1.02±0.15
5/Male	3	Castrated	11 KA	Short day	1.77±0.11	2.05±0.41
*Experiment 4: ERG test*						
5/Male	3	Sham-operated	Empty	Long day	2.21±0.20	2.91±0.33
5/Male	0	Castrated	Empty	Long day	1.89±0.07	1.18±0.29
5/Male	5	Castrated	11 KA	Long day	1.99±0.10	3.12±0.41
5/Male	0	Sham-operated	Empty	Short day	2.10±0.18	1.26±0.54

11 KA: 11-ketoandrostenedione implants; Empty: empty implants; GSI: gonadal somatic index in females; KSI: kidney somatic index in males. Short day (L8D16) and long day (L16D8) photoperiod.

Experiments 1–3 were performed with the permission of the Stockholm Northern Animal Experiment Ethical Committee (N 174/11). Experiment 4, which was conducted in Taiwan, was performed with permission (RFiZOOYH20060701) from the Institutional Animal Care and Use Committee (IACUC) of Academia Sinica.

### Experiment 1. Sexually mature and immature fish

Male and female sticklebacks were moved to 200 L aquaria and kept under long day (L16:D8) or short day photoperiods (L8:D16) for 28 or 32 days, respectively. At the end of the experiment (Apr 17^th^ 2012 for males, May 11^th^ 2012 for females) most of the fish kept under long day conditions had matured, whereas all fish kept under short day conditions remained immature. Males that showed breeding colours, i.e. blue irises and a bright red belly [12], were selected for retinae dissection. Sexually mature females were identified by their positive reaction [29] to the mating dances of a nesting male in a 50 L aquarium. Fish maintained under a short day (L8:D16) photoperiod at 20°C all remained immature.

### Experiment 2. Gonadectomy under long photoperiod

On May 21^st^ 2012, sexually immature fish were anaesthetized with 0.025% buffered MS-222 (Ethyl 3-aminobenzoate, methanesulfonic acid salt), and gonadectomies were performed by making ca. 1.5 mm long incisions into the abdominal cavity on each side, and excising the testes with fine forceps. Sham-operations were performed similarly, but the testes were not removed. The incisions were closed with BV-2 (0.4 Ph. Eur) sutures. After surgery, the fish were maintained under L16:D8 in a 700 L aquarium and dissected after 28 days.

### Experiment 3. Castration and androgen treatment

Implants (5 mm in length) were made from medical grade Silastic tubes (inner diameter (ID): 0.64 mm; outer diameter (OD): 1.19 mm), filled with crystalline 11 KA (4-androstene-3,11,17-trione; Sigma), and sealed with silicone glue. The hormone 11 KA is a non-aromatisable androgen, which is converted outside of the testes to 11-ketotestosterone (11 KT) in fish. In a previous study [30], similar 11 KA implants increased plasma 11 KT levels in castrated male sticklebacks to c. 225 ng/mL, slightly lower than levels observed in breeding, nesting males (c. 300 ng/mL). Empty implants were used as controls.

After surgery, the males were maintained under long or short day photoperiods in 200 L aquaria, and dissected after 28 days between May 23^rd^–26^th^. In earlier studies, changes in opsin expression occurred after even shorter periods (c. 3 weeks) of treatment in three-spined stickleback, salmonids, and goldfish [18], [31], [32].

### Experiment 4. Electroretinogram (ERG) recording

Fish were sent by airfreight to Taiwan in July 2013. After arrival, males were kept under a short day photoperiod at 20°C for 2 weeks before experimental treatments. All operations were performed as described above. Five castrated males, 5 castrated males with 11 KA implants, and 5 sham-operated fish were maintained separately in 30 L aquaria under a long day photoperiod at 20°C for 28 days. Five sham-operated males were kept under short day conditions.

Light-adapted fish were immobilized by injection of gallamine triethiodide (Flaxedil; Sigma, St. Louis, Mo. USA.), a neuromuscular junction blocking agent, into the dorsal muscle (0.4–0.6 µg per gram of body weight) after being anaesthetized with MS-222. Treated fish were wrapped in wet KimWipes (Kimberley-Clark Taiwan, Taipei) to prevent scraping or drying of the skin. Small pieces of wet KimWipes were used to cover the edges of the eye, to keep it wet without blocking the pupil. A small tube inserted into the mouth was used to irrigate the gills with oxygenated water during the recordings. Immobilized fish were kept under diffuse white background light (c. 12 lx) [33] for 1 hr before the ERG test. The rationale for using light-adapted fish was to obtain results comparable with data obtained from optomotor studies [4], [14].

ERG tests were performed as described previously [33], [34]. A 100 W halogen lamp (Osram 64637 100 W/12 V) was used as the light source in a lamp housing device (Nikon C-FI 115, Japan). Nine narrow bandpass filters (Asahi Spectra, Japan) provided light stimuli at wavelengths of 400, 440, 460, 480, 500, 520, 560, 600, and 670 nm. The full width at half maximum of the bandpass filters was 10±2 nm (400–560 nm) or 12±2 nm (600 and 670 nm). A series of neutral density (ND) filters (Andover, NH, USA) were used to reduce the light intensity in 0.3 log unit steps, from −0.3 to −4.8 log units (Fig. S1). Peak photon fluxes at each wavelength were determined using a USB 2000 spectrometer (Ocean Optics, USA), calibrated by LC-1-cal (Ocean Optics, USA) with OODBase32 software (Ocean Optics, USA), at the fish eye. A shutter (Sutter Instrument Lambda SC, USA) was used to provide fixed durations of light stimuli (10 stimuli, each of 20 ms in duration, with an inter-stimuli interval of 5 s) in a diffuse white background (12 lx) [33], i.e., under a light-adapted condition. The ERG tests started from the lowest light stimuli, and ND filters were adjusted step-by-step to increase stimuli intensity (15 steps to reach the maximum). ERG signals were recorded using Teflon-coated insulated silver electrodes (63.5 µm in diameter) with exposed chlorinated tips. The recording electrode was positioned on the cornea and the reference electrode was placed on the head. The signals from both electrodes were amplified 20 k-fold and filtered by a high-low band pass filter set between 1 and 3000 Hz (ER-1, Cygnus Technology Inc. USA). The signals were digitized at a 2 kHz sampling rate with PowerLab 4/25 (ADInstruments, Inc. ML845, Australia), and recorded with Scope 3 data acquisition software (ADInstruments, Inc. Australia).

**Figure 1:**
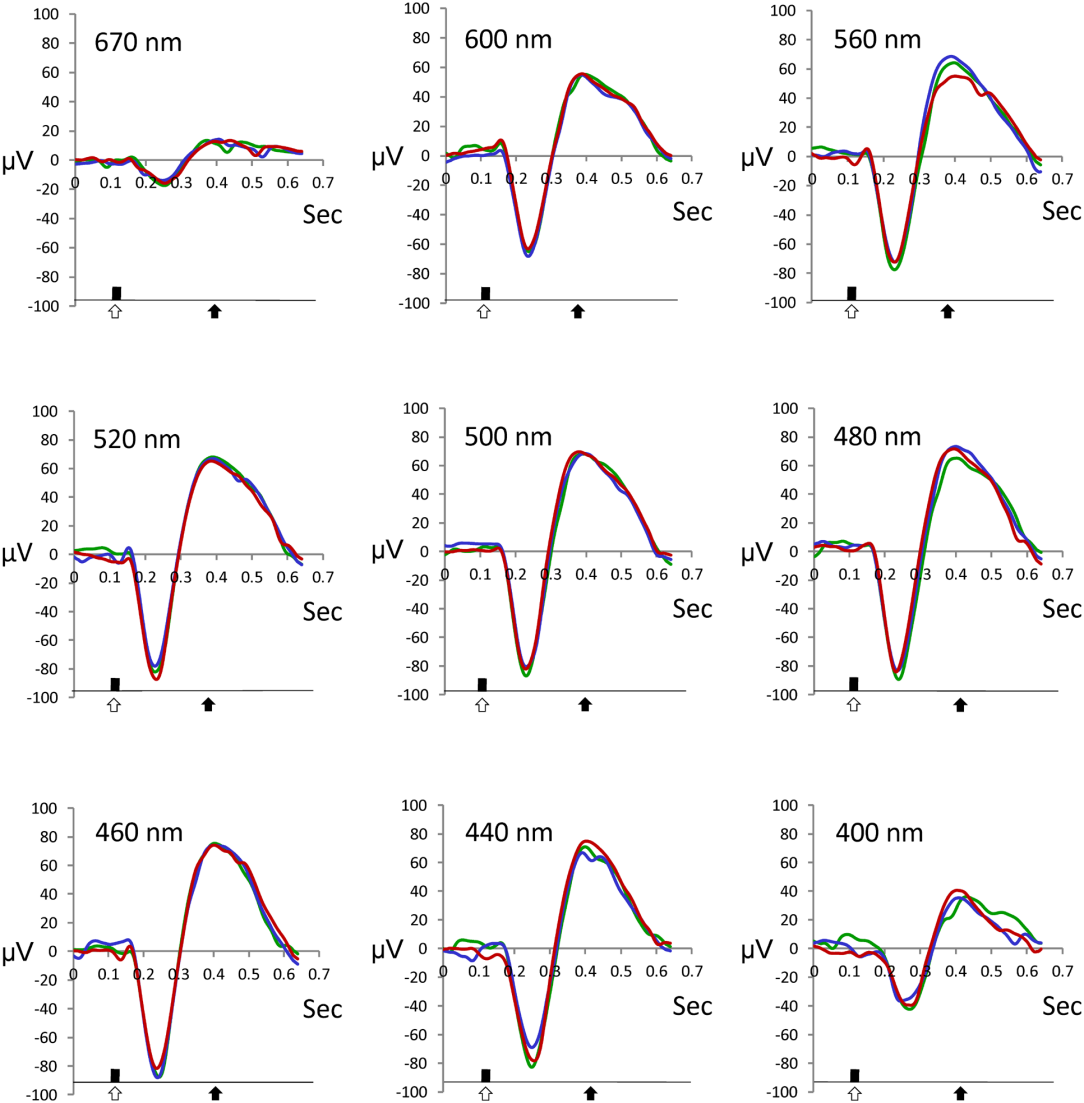
ERG traces of sticklebacks. Typical ERG traces taken from maximum light stimuli (step 15) for each spectrum. Three repeats are shown. Open arrows indicate the stimuli and solid arrows indicate the presence of *b*-waves.

The recorded signals were analyzed using custom-made software (programmed by K. Anraku). Signals recorded at each step (100–700 ms after stimuli) were compared to the model ERG trace obtained at maximum light stimuli using the correlation method (Fig. 1). The replicates of waveforms from each test were compared using the Spearman correlation test. Our pilot study revealed that when a correlation coefficient (*r*) between two replicates was less than 0.7, the two ERG traces showed little resemblance. Hence, ERG traces were considered valid when *r* was greater than 0.7, and the *b*-wave time delay was less than 100 ms [34]. Deflection of the ERG trace was taken as an indication of the light intensity threshold of the particular wavelength [33]. Sensitivity was defined as –log_10_ of the threshold for each test wavelength. To remove variations between tests, values for each fish were normalized to a 0–100% scale, where 100% indicated the maximum sensitivity spectrum [35].

### Dissection

The fish were dissected at around 1 pm to 3 pm, which was during the middle of the day time for both photoperiods. All fish were euthanized with 0.025% buffered MS-222 solution before dissection under a stereomicroscope. Body, ovary, and kidney weight were measured, and the presence of breeding colours was noted. Kidney weight was used as an indicator for male maturation, as the kidneys of breeding male sticklebacks undergo hypertrophy upon androgen stimulation, and begin to produce the protein spiggin [36] for use in nest-building. The Gonad-Somatic Index (GSI) and Kidney-Somatic Index (KSI) were calculated as (organ weight/body weight) *100. All castrations were found to be complete (no remnants) and all implanted capsules were present. Retinae were removed from eyes and immersed in 500 µl RNAlater (Ambion) on ice for 2 hours, before being stored at −80°C until further analyses.

### Retinal RNA Extraction and cDNA Synthesis

Total RNA was extracted using the RNeasy Lipid Tissue Mini Kit (Qiagen cat. 74804) and reverse transcribed using Super-Script III (Invitrogen cat. 18080-400), according to the respective manufacturer's protocols. Possible genomic DNA contamination was removed by treating RNA samples with DNase (2 U) from the TURBO DNA-free kit, according to the manufacturer's instructions (Ambion).

### PCR

The whole genome sequence of three-spined stickleback has been published on the UCSC Genome Bioinformatics Site, and five retinal opsin genes, *rh1*, *rh2*, *sws1*, *sws2*, and *lws*, were identified by gene prediction. Gene-specific primers (Table 2) were designed based on the predicted genomic opsin sequences, and were used to amplify the opsin genes from the retinal cDNA of three-spined stickleback. The PCR products were obtained using Fast-Run Taq Master Mix (Protech Technology Enterprise Co., Taiwan) in 20 µl reactions, in accordance with the manufacturer’s recommended reaction concentrations. Each reaction was performed using an ABI Veriti Thermal Cycler (Applied Biosystems, California, USA) and the following steps: 35 cycles of denaturation at 95°C for 30 sec, annealing at 50°C for 40 sec, extension at 72°C for 1 min; and a final extension step at 72°C for 5 min. The PCR products were then purified using the Qiagen purification kit, subcloned into the pGEM-T Easy vector (Promega; Madison, WI), sequenced, and identified. The sequences of opsin genes were uploaded to the NCBI database: *rh1* (NCBI: KC774627), *rh2* (NCBI: KC774624), *sws1* (NCBI: KC774626), *sws2* (NCBI: KC774625), and *lws* (NCBI: KC774623).

**Table 2:** Sequences of the primers used for PCR and *q*-PCR.

Gene		Forward primer	Reverse primer
*lws*	PCR	5′-ATGGCAGAAGAGTGGGGAAAGC-3′	5′-TTATGCAGGAGCCACAGAGGAG-3′
*E_i_* = 95.4	*q*-PCR	5′-CCTGGGAGAGATGGATAGTTGTGT-3′	5′-CCTGGGAGAGATGGATAGTTGTGT-3′
*rh2*	PCR	5′-ATGGCCTGGGAAGGAGGACTCG-3′	5′-TTAAGACACAGAGGACACTTCTGTC-3′
*E_i_* = 93.1	*q*-PCR	5′-ACCATCACGTCGGCTGTCA-3′	5′-TGGCCATGAATCCCTCAAG-3′
*sws1*	PCR	5′-ATCACGATGGGGAAACACTTCC-3′	5′-AAGAAGCTGTGGACACTGATGAC-3′
*E_i_* = 94.2	*q*-PCR	5′-CTCGTCACAGCCAAATACAAGAA-3′	5′-AATCCTGCCAAGGTGATGTTG-3′
*sws2*	PCR	5′-ATGAAGCACGGCCGCGTCCCAG-3′	5′-CTAAGCAGGTCCAACTTTGG-3′
*E_i_* = 94.7	*q*-PCR	5′-GCGGTCCCACCTCAACTACA-3′	5′-CGGACACGAGAAGGTTTGACA-3′
*rh1*	PCR	5′-ATGAACGGCACAGAGGGACCCT-3′	5′-TTACGCGGGAGACACGGAGCTGG-3′
*E_i_* = 99.1	*q*-PCR	5′-CGCCGCCCAGCAGGAGT-3′	5′-GCGTAGGGCACCCAGCACAC-3′
*β-actin*			
*E_i_* = 104.2	*q*-PCR	5′-CCAAAGCCAACAGGGAGAAGATG-3′	5′-GGCGTACAGGGACAGCACAGC-3′

Efficiency (*E_i_*) (%) for q-PCR primers.

### q-PCR

The specific primers designed for q-PCR were based on the sequences identified by PCR. The amplification efficiency and melting curve of each q-PCR primer pair was tested by 10-fold serial dilutions of the templates (100 pg to 100 ng), with 3 replicates for each gene and sample. Each reaction contained 40 ng of cDNA, 50 nM of each primer (Table 2), and LightCycler 480 SYBR Green I Master Mix (Roche) in a final volume of 10 µl. Each reaction was performed using a Roche LightCycler 480 System (Roche Applied Science, Mannheim, Germany) with the following steps: 1 cycle of 50°C for 2 min and 95°C for 10 min, followed by 45 cycles of 95°C for 15 sec and 60°C for 1 min. PCR products were subjected to a melting-curve analysis, and representative samples were electrophoresed to verify that only a single product was present. Control reactions were conducted with RNA-free water to determine background levels. Three replicates were performed for each gene/sample.

Expression of each opsin gene was normalized against that of the *β-actin* reference gene, or determined as a fraction of the total cone opsin gene expression in an individual following the equation of Carleton and Kocher (2001) [37], which has been modified in Fuller et al. (2004) [38] and Spady et al. (2006) [39].
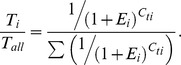
where *T_i_/T*_all_ is the relative gene expression ratio for a given gene normalized by total cone opsin gene expression, *E_i_* is the PCR efficiency for each pair of primers, and *C_ti_* is the critical cycle number for each gene. Proportional opsin mRNA levels are given as percentages of total cone opsin gene expression.

### Statistics

Data were analysed using SPSS v. 14. Data from two groups were compared by performing the Shapiro-Wilk normality test (p>0.05), followed by two-tailed Student’s t-test. Multiple group comparisons were performed by one-way ANOVA, followed by post hoc analysis with Tukey’s test, i.e. exp. 3. Moreover, two-way ANOVA was used to analyze the effects of multiple factors on the opsin mRNA levels in exp. 1 and 3.

## Results

### General

Castrated males were observed to have lower body weights than sham-operated males (exp. 2, 3, and 4), but otherwise no significant differences in body weight were observed between experimental groups of the same sex (Table 1). The KSI and GSI values were higher in sexually mature than in immature fish, for both males and females (exp. 1). Most sham-operated males kept under long day conditions exhibited clear breeding colours, and higher KSIs than castrated males (exp. 2, 3 and 4). However, neither sham-operated nor castrated control males displayed breeding colours under short day conditions (exp. 3). Under both long and short day conditions, 12 of 15 of the castrated males treated with 11 KA exhibited breeding colours; furthermore, the KSIs of castrated fish treated with 11 KA were higher than those of castrated control fish (exp. 3 and 4; Table 1), and KSIs were slightly higher in the former maintained under long photoperiods than equivalents under short photoperiods (p = 0.05).

### Opsin mRNA levels

**Figure 2:**
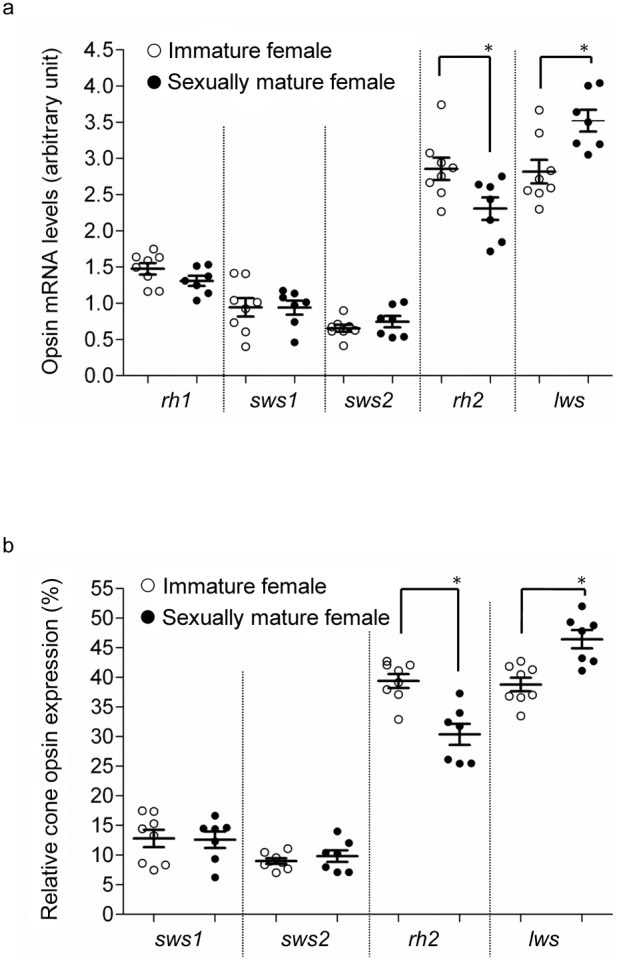
Opsin mRNA levels in sexually mature and immature females. Experiment 1; (a) Relative opsin mRNA levels and (b) proportions of cone opsin mRNA levels in immature (○) and sexually mature (•) females. Means ± SEM are shown. *p<0.05.

**Figure 3:**
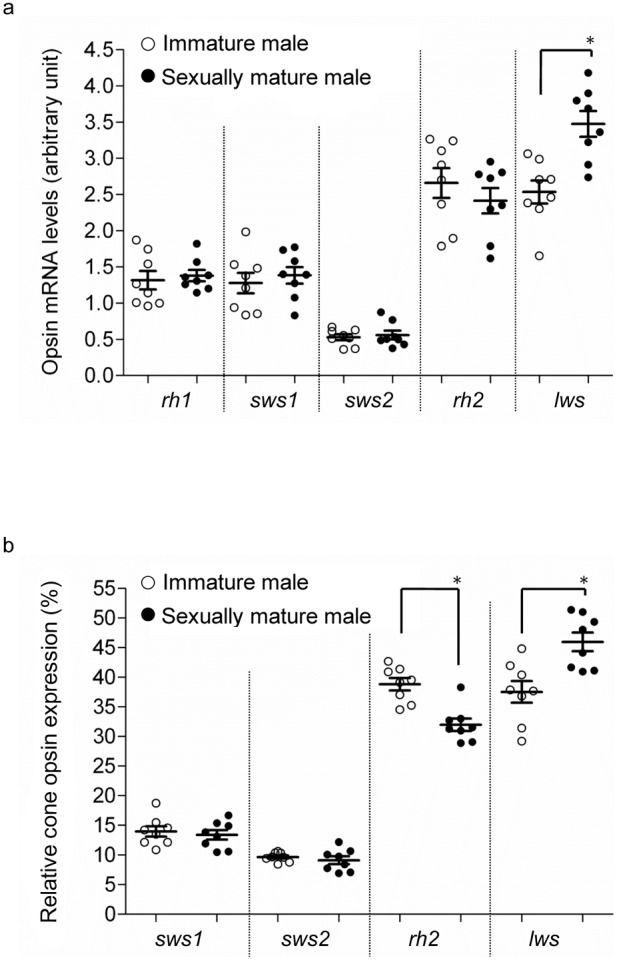
Opsin mRNA levels in sexually mature and immature males. Experiment 1; (a) Relative opsin mRNA levels and (b) proportions of cone opsin mRNA levels in immature (○) and sexually mature (•) males. Means ± SEM are shown. *p<0.05.

In exp. 1, levels of *lws* mRNA (relative to *β-actin*) were significantly higher in sexually mature than in immature fish, in both females (Fig. 2a) and males (Fig. 3a). Relative *rh2* mRNA levels were higher in immature females than in sexually mature females, but no significant difference was observed in males (p = 0.09) (Fig. 2, 3). Furthermore, the level of *lws* mRNA as a proportion of all opsins was higher in sexually mature than in immature fish (for both males and females), whereas proportional *rh2* mRNA levels were lower in sexually mature fish (Fig. 2b, 3b). No differences were observed in the proportions of the mRNA levels of other opsin genes (Fig. 2, 3). While reproductive condition (i.e., sexual maturity) significantly affected relative and proportional *lws* and *rh2* mRNA levels (two-way ANOVA, p<0.05 in all comparisons) in stickleback, sex was not observed to have an effect on either relative or proportional expression of *rh2* or *lws* (two-way ANOVA, *rh2*: p = 0.804 or 0.820; *lws*: 0.603 or 0.581).

**Figure 4:**
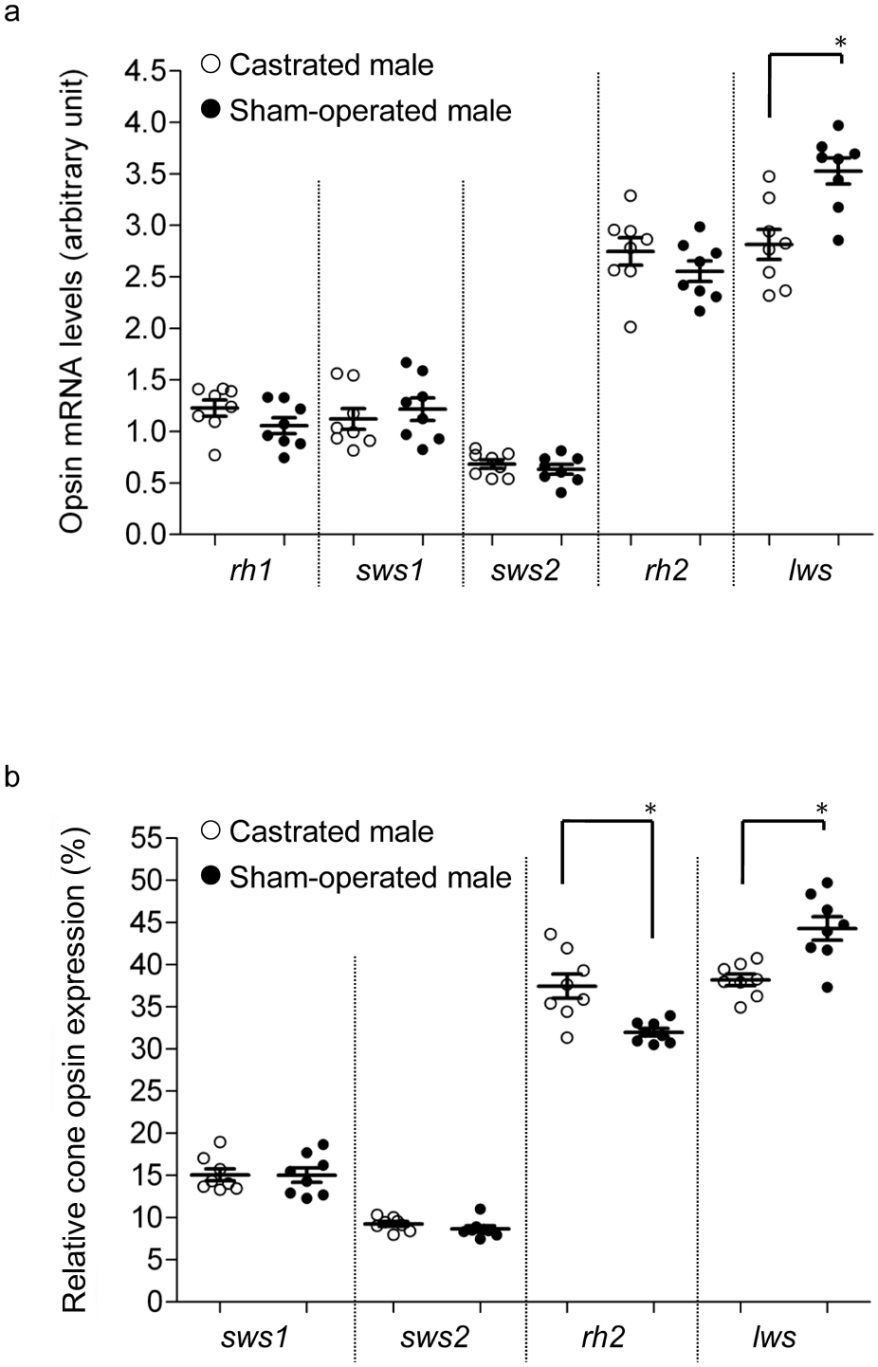
Opsin mRNA levels in castrated and sham-operated males. Experiment 2; (a) Relative opsin mRNA levels and (b) proportions of cone opsin mRNA levels in castrated (○) and sham-operated (•) males. Means ± SEM are shown. *p<0.05.

Through exp. 2, we observed that relative levels of *lws* mRNA under long day conditions were higher in sham-operated males than in castrated males, while mRNA levels of other opsin genes, including *rh2*, were unchanged (Fig. 4a). Sham-operated males exhibited a higher proportional level of *lws*, but a lower proportional level of *rh2*; mRNA levels of other opsin genes were unaffected (Fig. 4b).

**Figure 5:**
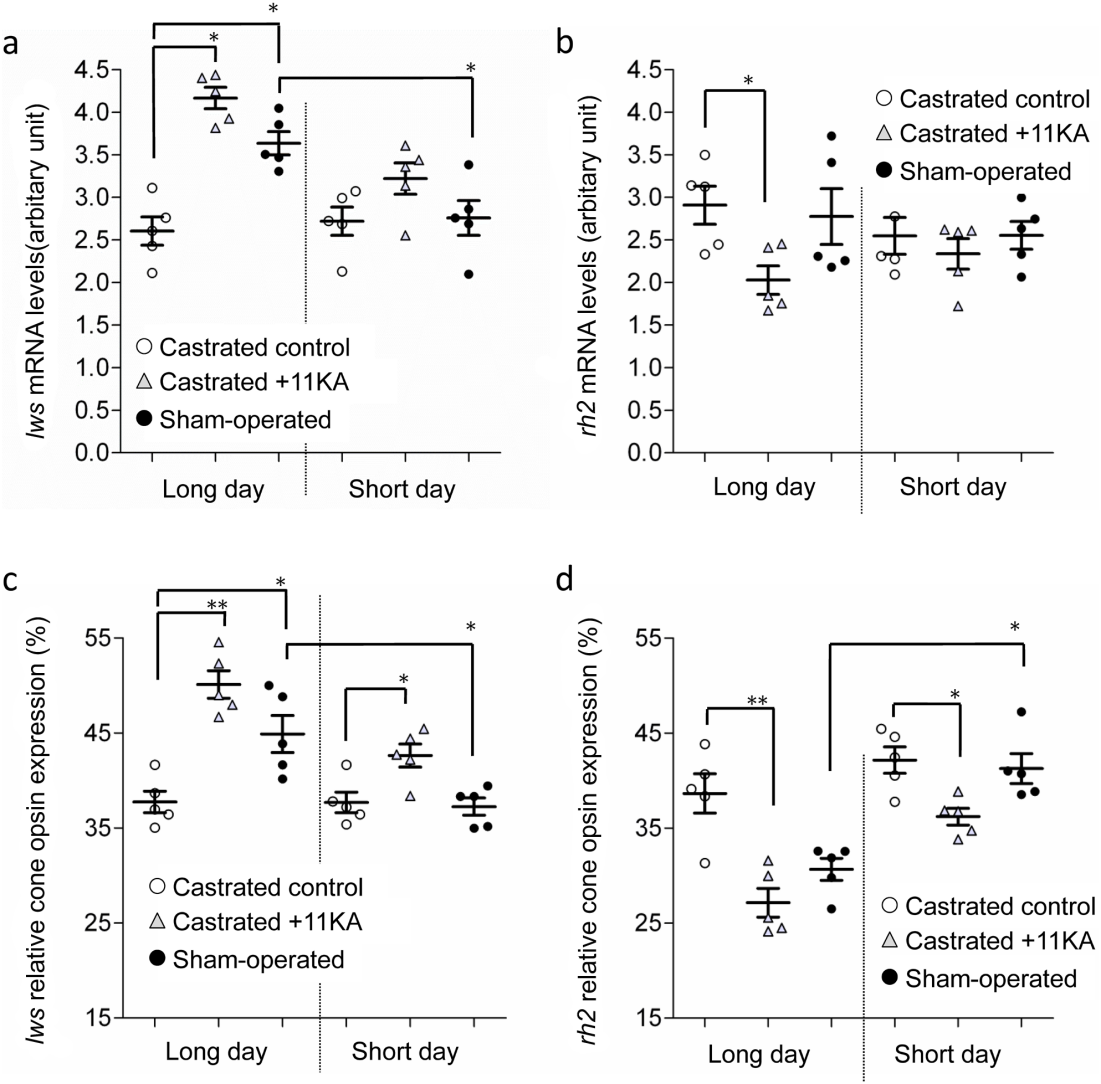
Opsin mRNA levels in castrated males treated with androgen. Experiment 3; effects of castration and androgen treatment on relative (a) *rh2* and (b) *lws* mRNA levels, and (c) *rh2* and (d) *lws* levels as a proportion of total cone opsin mRNA, under long or short day conditions. Castrated control males (○), castrated males with 11 KA treatment (▵), and sham-operated males (•). Means ± SEM are shown. *p<0.05; **p<0.005.

In exp. 3, sham-operated males kept under long day conditions exhibited higher relative *lws* mRNA levels (Fig. 5a) and higher proportional *lws* expression (Fig. 5c) than those kept under short day conditions. Under long day conditions, castrated males with 11 KA implants exhibited higher relative levels of *lws*, but lower levels of *rh2* mRNA, than castrated control males (Fig. 5a, b). However, under short day conditions, significant differences between these groups were only observed for proportional *lws* and *rh2* levels (Fig. 5c, d). 11 KA significantly affected relative and proportional *lws* mRNA levels (p<0.001) and proportional *rh2* mRNA levels (p<0.05) in castrated fish under both photoperids (Two-way ANOVA). Furthermore, photoperiod was found to affect proportional *lws* mRNA levels (p<0.001), but not relative or proportional *rh2* mRNA levels (p = 0.461; p = 0.285). The relative and proportional mRNA levels of the other opsin genes were not affected by castration or 11 KA treatment, under either short or long day photoperiods.

### ERG

**Figure 6:**
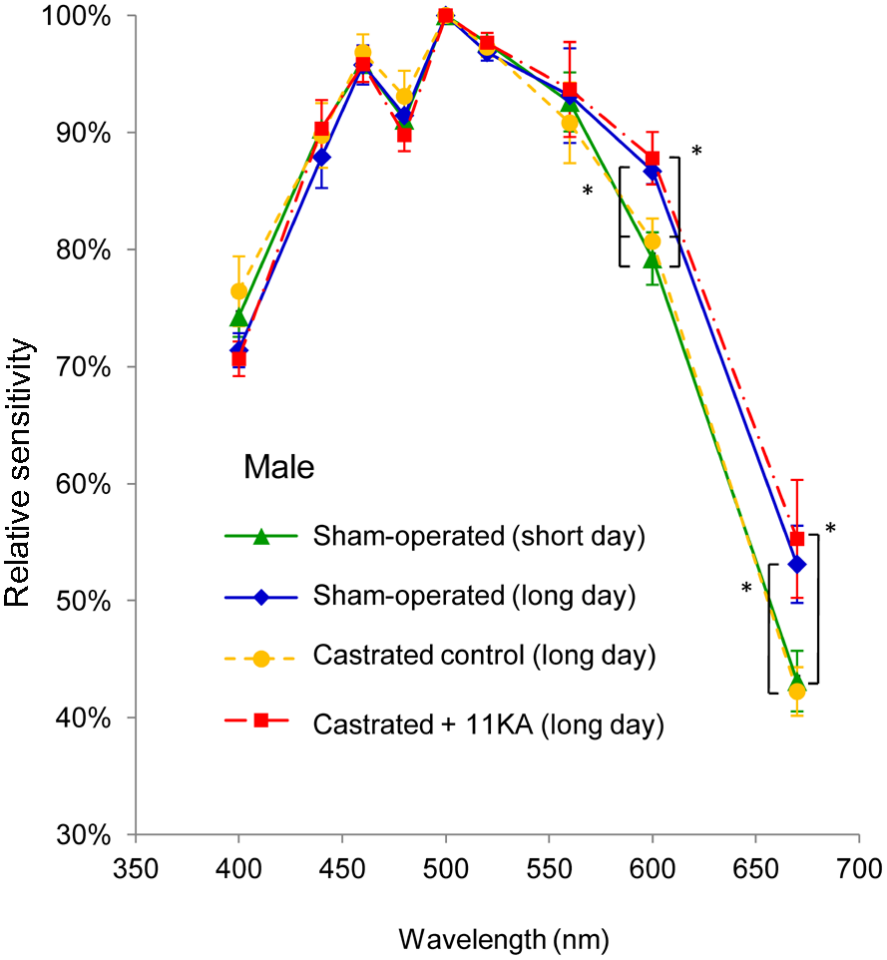
ERG spectral sensitivities. Experiment 4; spectral sensitivities of sham-operated males, castrated control males, and castrated males treated with 11-ketoandrostenedione under long day photoperiods, and sham-operated males under a short day photoperiod. Spectral sensitivities were determined through ERG analyses of 5 fish for each group. All data were normalized to the threshold at the most sensitive wavelength (i.e. 500 nm) for each fish. Means ± SEM are shown. *p<0.05.

Light-adapted sticklebacks exhibited the greatest sensitivity (lowest threshold) to light with a wavelength of 500 nm (21.5±1.56 quantum flux (10^−5^ µmol/m^2^/s); means ± SD) (Fig. S2). No significant difference in the threshold at 500 nm was observed between treatments (p>0.05 for each comparison). Figure 5 shows the relative spectral sensitivity curves, which were normalized to the sensitivity at 500 nm for each fish. The relative sensitivities in the green spectrum (500 or 520 nm) were far higher than those for red (600/670 nm) and blue (440 nm) for all treatments. Moreover, male sticklebacks were more sensitive to short wavelength red light (600 nm) than long wavelength red light (670 nm) (Fig. 6).

In exp. 4, sham-operated males maintained under long day photoperiods exhibited greater sensitivity to light with a wavelength of 600 nm than those maintained under short day conditions, whereas no significant difference in sensitivity to 670 nm light was observed (p = 0.073). Under long day conditions, sham-operated males and castrated males treated with 11 KA showed significantly greater relative sensitivity to the red part of the spectrum (600 and 670 nm), as compared to castrated control males (Fig. 5). No significant difference in relative sensitivity was observed at any other wavelength.

## Discussion

Breeding colours and high KSIs were observed in males kept under long day, but not under short day conditions, in agreement with the findings of several earlier studies [40]–[42]. Both traits were suppressed by castration and stimulated by 11 KA, also consistent with previous reports [41], [42]. Levels of *lws* mRNA were higher in sexually mature than in immature male and female sticklebacks. Moreover, the ERG results revealed that short photoperiod males were less sensitive to red light (at least at 600 nm) than males stimulated to breed by long photoperiod. In addition to the findings of previous optomotor test experiments [4], [14], the molecular and electrophysiological results confirmed that the red light sensitivity of stickleback changes during the breeding period. Furthermore, our current results are consistent with the reported increase in red light sensitivity exhibited by sticklebacks of both sexes in summer [4]. Changes in spectrum sensitivity are often due to changes in the photic environment [43], [44], rather than changes in physiological conditions. However, the effects of castration and androgen-treatments reported here indicate a specific role in reproductive physiology, most likely an enhanced sensitivity to the male signal. The observed correlation between *lws* mRNA levels and red light sensitivity described here are in general agreement with findings in zebrafish [23], in which circadian rhythms of behavioural red light sensitivity are correlated with *lws* expression.

Despite reduced levels of *rh2* mRNA in sexually mature male sticklebacks and androgen-treated castrated males, ERG (this study) and optomotor tests [14] failed to reveal any changes in green light sensitivity. Since the λ_max_ of the stickleback rod photopigment overlaps with the spectral range of *rh2* opsin [18], the ERG signals at wavelengths 500 to 540 nm potentially represent the overlap of rod and *rh2* cone signals, even if the fish were adapted to light. Hence, the ERG thresholds at these spectral ranges may not be representative of actual green colour vision.

Our results demonstrate that exposure to long day photoperiods fails to increase *lws* mRNA levels in castrated males. However, androgen treatment had a distinct stimulatory effect on both *lws* mRNA level and ERG red light sensitivity in castrated males. Although a connection between androgens and the visual system has not been previously demonstrated, sex steroids are known to enhance pheromone sensitivity in salmon [8]. In juvenile tinfoil barb (*Puntius schwanenfeldii*), olfactory sensitivity to prostaglandin (a fish sex pheromone) is increased at 9 days after treatment with 17α-methyltestosterone [45]. Moreover, treatment with 17β-oestradiol and testosterone can up-regulate pheromone receptor genes (VR1 and VR4) in the basal zone of the vomeronasal organ in mouse [9].

Several mechanisms may alter visual sensitivity in fishes, including changes in chromophore types [19], changes in the assembly of photoreceptors, and altered opsin levels within a photoperiod class [46]–[48]. In stickleback, different proportions of double cone types (red/red; green/red) have been detected in fish collected from waters with different levels and compositions of ambient light [18]. However, the overall single and double cone mosaics are largely consistent between fish. In sticklebacks transferred from red light-shifted lakes to clear water, the proportion of green/red double cones increased, while the proportion of red/red double cones decreased slightly; on the other hand, double cone density remained constant [18]. In the current study, we detected changes in *lws*/*rh2* expression, but no significant changes in relative or proportional *sws1*/*sws2* mRNA levels, suggesting that breeding-induced shifts in spectral sensitivity may arise from double cones, but not from single cone assembly. Such changes in *lws*/*rh2* proportion may be a result of green/red double cone apoptosis and their replacement by newly differentiated red/red double cones [18], or simply by changes in opsin content in an existing cone.

Effects of hormones or neurotransmitters on opsin gene expression have been shown previously in some fishes, although these have not been found to be closely related to reproductive physiology. In coho salmon (*Oncorhynchus kisutch*), spectral sensitivity changes during smoltification when fish are preparing to migrate from fresh water to the sea [49]. Administration of L-thyroxine (T_4_), which increases naturally during smoltification [50], results in up-regulation of the *rh2a* opsin gene, and may thus induce this visual sensitivity shift before migration [49]. In zebrafish, dopamine (DA) treatment increases levels of *lws* opsin mRNA, which possibly results in higher red light sensitivity during the daytime [23]. Furthermore, orally-administered gonadotropin-releasing hormone (GnRH) can up-regulate *rh2b* and down-regulate *rh2a* mRNA levels in the retinae of zebra cichlids (*Metriaclima zebra*) [22]. The best-known role of GnRH is that it stimulates reproduction via gonadotropin release, but it also has many other roles [51]. GnRH-immunoreactive fibres innervate the retinae of several fishes, including the stickleback [52].

In addition to altering opsin expression, hormones can also change A1/A2 chromophore types. Injection of European eel (*Anguilla anguilla*) with human chorionic gonadotropin (HCG) and salmon pituitary extract can shift the peak sensitivity wavelength of retinal rod cells [21]. Treatment of juvenile rainbow trout with T_4_ induced not only an opsin expression switch, but also a chromophore shift from vitamin A1 to vitamin A2 [53]. Although the effects vary among species and physiological conditions, T_4_ and 3, 5, 3′-triiodo-L-thyronine (T_3_) may interact with the reproductive endocrine system [54], [55]. The results of these earlier studies suggest that switching between the A1 and A2 chromophores may be another mechanism of fine-tuning spectrum sensitivity.

Both sexes display a similar increase in red light sensitivity [4] and *lws* opsin mRNA levels in the breeding state. The present study indicates that androgens, which control reproductive behaviour and secondary sexual characteristics in stickleback [42], [56], [57], also control red light sensitivity in the male. In the male stickleback, 11 KT is the most abundant and effective androgen, and its levels peak in the breeding period [28]. Although 11 KT levels in female sticklebacks are much lower than in males, T levels are higher, and also peak in the breeding period [28]. Red light sensitivity in the female may, therefore, also be controlled by androgens (or other gonadal hormones), although it was not possible to test this adequately as females cannot be gonadectomised. Though it is clear that androgens control red light sensitivity in male sticklebacks, the exact mechanism requires further elucidation. Androgen may have direct effects in the retinae [26], or its effect may be exerted via other neuronal or hormonal systems.

The ability of female sticklebacks to detect the red breeding colour of males is not trivial. In eutrophic environments with turbid water, spectral signalling becomes less effective. Females are more likely to choose males in clear water (which are more visible) than males in turbid water, even if the former are less red [58]. A female with a reduced capability to detect red would be less able to observe males. This is particularly significant, as it is not just a question of a female stickleback choosing the best possible mate, but also of getting a mate at all, as it is common for female sticklebacks to court the male, especially at the end of the breeding season [59]. A male with a high sensitivity to red light during the breeding period would have a selective advantage in knowing the whereabouts of his rivals, to defend his territory and prevent sneaking [6], [7], which in the stickleback is largely a behaviour exhibited by other territorial, also red, males.

In conclusion, the relative and proportional levels of *lws* mRNA increase in both sexes during sexual maturation, which is consistent with the higher red light sensitivity in the breeding state. In male sticklebacks, the changes in *lws* mRNA and red spectral sensitivity are dependent on androgen, indicating that the changes in the retina are specifically related to reproductive physiology.

## Supporting Information

Figure S1 **Photon fluxes at different wavelengths.** A series of neutral density (ND) filters were used to reduce the light intensity in 0.3 log unit steps from −0.3 to −4.8 log units (15 steps). Narrow bandpass filters provided light in the wavelength range 400–670 nm.
(TIF)

Figure S2 **ERG spectral threshold.** Spectral threshold of sham-operated males, castrated control males, and castrated males treated with 11-ketoandrostenedione under long day photoperiod, and sham-operated males under a short day photoperiod. Spectral thresholds were determined through ERG analyses of 5 fish for each group. A logarithmic scale is used for the y axis. Means ± SEM are shown.
(TIF)
