# Supplementary figures and images for: Androgens Increase lws Opsin Expression and Red Sensitivity in Male Three-Spined Sticklebacks
Source: PLoS One. 2014 Jun 25;9(6):e100330. doi: 10.1371/journal.pone.0100330 (PMC4070989; doi:10.1371/journal.pone.0100330)

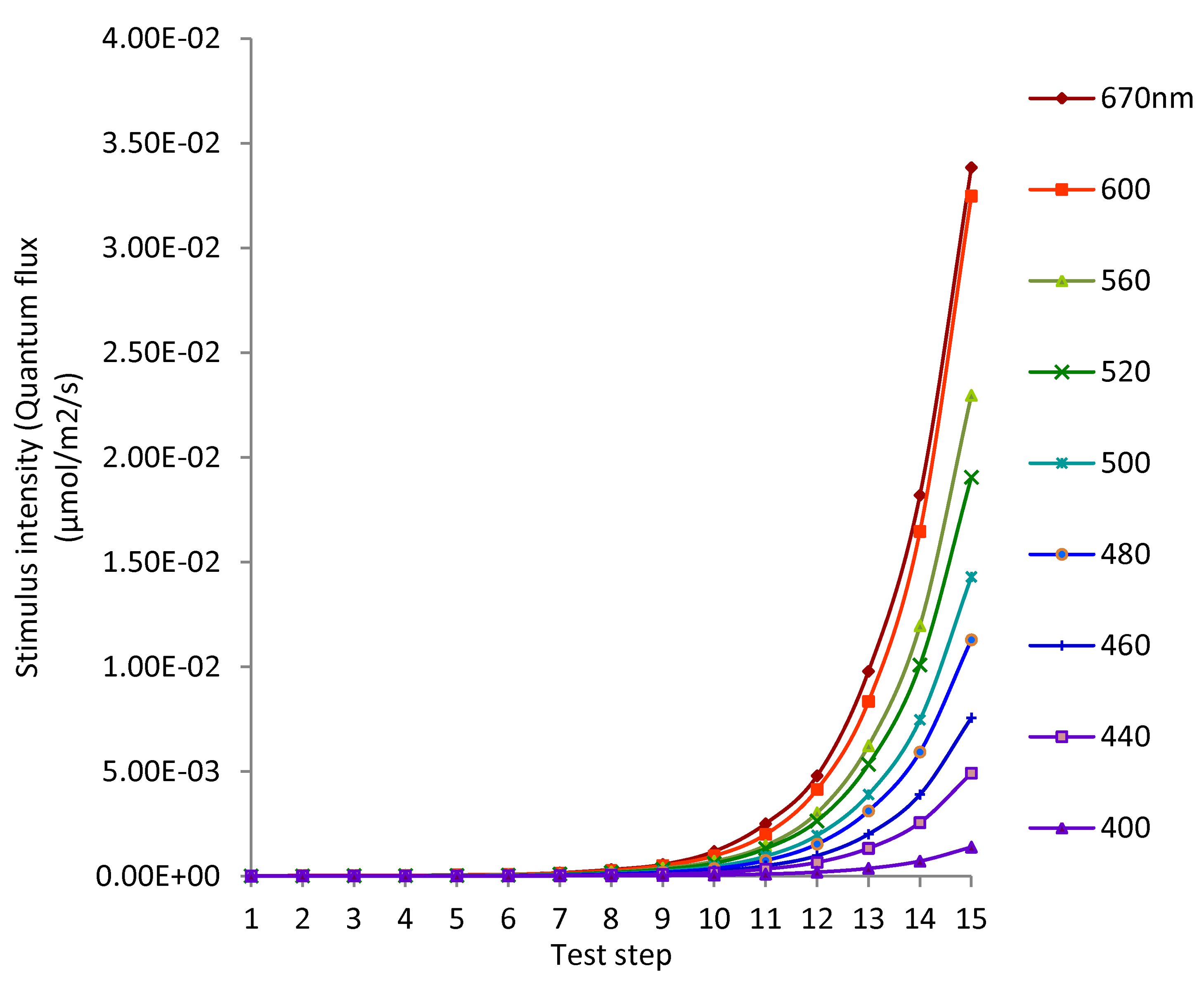

Supplement: Figure S1 — Photon fluxes at different wavelengths. A series of neutral density (ND) filters were used to reduce the light intensity in 0.3 log unit steps from −0.3 to −4.8 log units (15 steps). Narrow bandpass filters provided light in the wavelength range 400–670 nm. (TIF) [file pone.0100330.s001.tif]

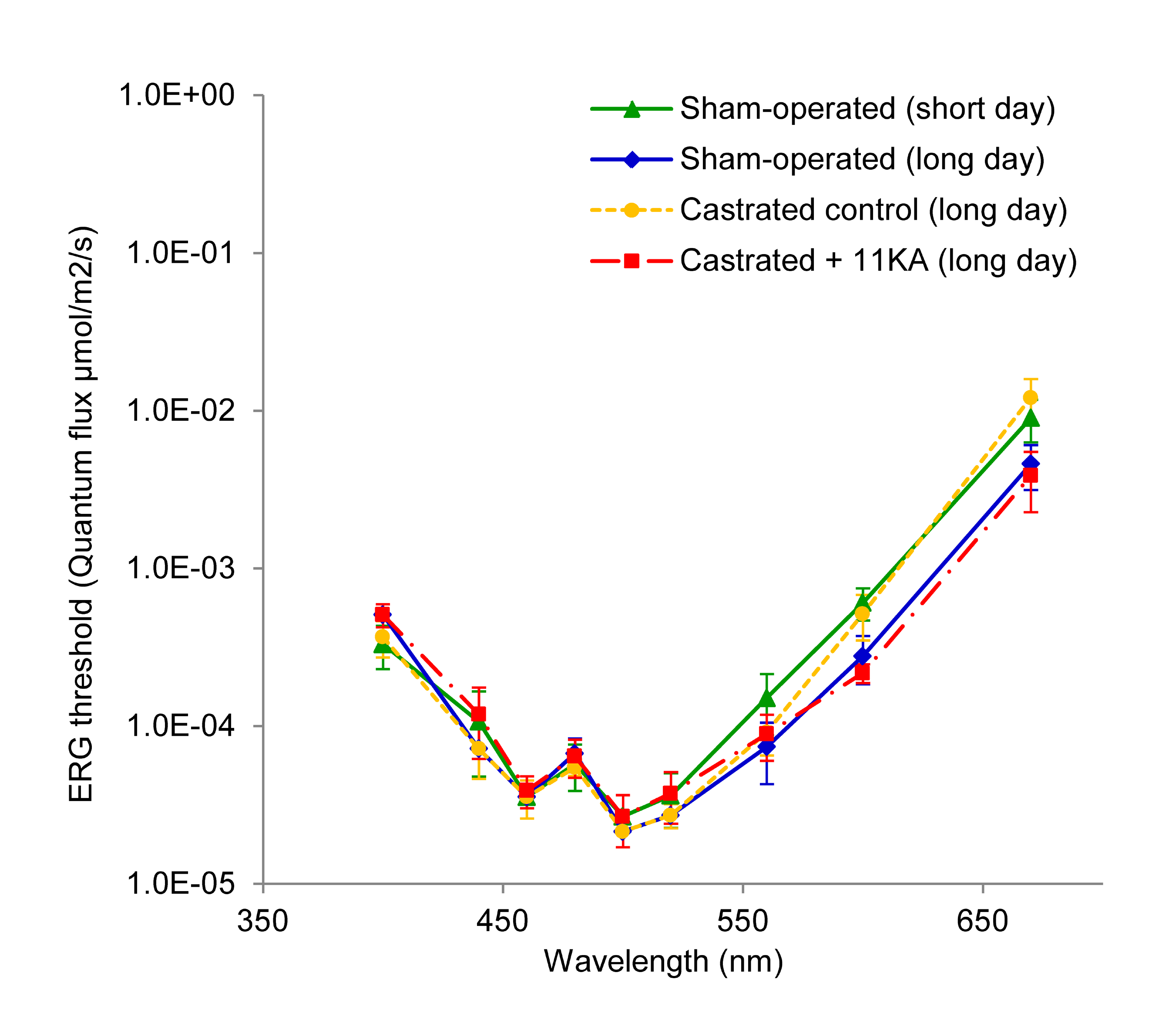

Supplement: Figure S2 — ERG spectral threshold. Spectral threshold of sham-operated males, castrated control males, and castrated males treated with 11-ketoandrostenedione under long day photoperiod, and sham-operated males under a short day photoperiod. Spectral thresholds were determined through ERG analyses of 5 fish for each group. A logarithmic scale is used for the y axis. Means ± SEM are shown. (TIF) [file pone.0100330.s002.tif]
